# Construction and analysis of protein–protein interaction networks based on nuclear proteomics data of the desiccation-tolerant *Xerophyta schlechteri* leaves subjected to dehydration stress

**DOI:** 10.1080/19420889.2023.2193000

**Published:** 2023-03-22

**Authors:** Ryman Shoko, Babra Magogo, Jessica Pullen, Reagan Mudziwapasi, Joice Ndlovu

**Affiliations:** a Department of Biology, Chinhoyi University of Technology, Chinhoyi, Zimbabwe; b Department of Animal Science and Rangeland Management, Lupane State University, Lupane, Zimbabwe; c Department of Research and Innovation, Midlands State University, Gweru, Zimbabwe

**Keywords:** Bioinformatics, hub proteins, protein–protein interaction, resurrection plants, Xerophyta schlechteri

## Abstract

In order to understand the mechanism of desiccation tolerance in *Xerophyta schlechteri*, we carried out an *in silico* study to identify hub proteins and functional modules in the nuclear proteome of the leaves. Protein–protein interaction networks were constructed and analyzed from proteome data obtained from Abdalla and Rafudeen. We constructed networks in Cytoscape using the GeneMania software and analyzed them using a Network Analyzer. Functional enrichment analysis of key proteins in the respective networks was done using GeneMania network enrichment analysis, and GO (Gene Ontology) terms were summarized using REViGO. Also, community analysis of differentially expressed proteins was conducted using the Cytoscape Apps, GeneMania and ClusterMaker. Functional modules associated with the communities were identified using an online tool, ShinyGO. We identified HSP 70–2 as the super-hub protein among the up-regulated proteins. On the other hand, 40S ribosomal protein S2–3 (a protein added by GeneMANIA) was identified as a super-hub protein associated with the down-regulated proteins. For up-regulated proteins, the enriched biological process terms were those associated with chromatin organization and negative regulation of transcription. In the down-regulated protein-set, terms associated with protein synthesis were significantly enriched. Community analysis identified three functional modules that can be categorized as chromatin organization, anti-oxidant activity and metabolic processes.

## Introduction

Resurrection plants can survive extreme water loss and survive long periods in an abiotic state and, upon watering, rapidly restore their normal metabolism [2]. Understanding the mechanisms of desiccation tolerance (DT) in resurrection plants is important as they are deemed to be an excellent model to study the mechanisms associated with the desiccation tolerance phenomenon.

Proteomics can generate expression information for the proteins in a sample at a given time point [3]. Thus, through proteomics, proteins that are up-regulated or down-regulated in response to perturbation are identified – the so-called differentially expressed proteins (DEPs) [4]. Proteomic profiling offers the opportunity to identify proteins that mediate the pathways involved in the DT mechanisms, when cells are subjected to desiccation stress. Several proteomics investigations have been carried out for the leaves of some angiosperm resurrection plants during desiccation [1], [5], [6], [7], [8], [9], [10]. However, in all these works, no attempt was made to gain deeper insights from the data using such bioinformatics approaches as protein–protein interaction (PPI) studies. PPI analysis can be used to carry out a rigorous investigation of proteomics data, producing novel insights and testable hypothesis [11], [12], [13], [14], [15], [16].

Generally, PPI studies have been extensively explored to investigate the biological significance of differential expression data and datasets, e.g. [17], [18], [19]. Given a list of proteins or genes, network biology algorithms make it possible to identify additional associated proteins [20]. This, arguably, is ideal when studying orphan species such as the resurrection plants. Often, high-throughput experiments in orphan plants do not lead to the identification of all proteins in a sample [9], [21], [22]. This is because protein IDs rely on inferring from homologs of distantly related species. In addition, analysis of biological networks makes it possible to identify what are called ‘hubs’. Hubs in a network are the highly connected nodes in a scale-free system and have come to be associated with the key genes in the networks [23], [24] Therefore, re-analysis of data deposited and stored in public databases or found in publications can provide valuable clues for new research. In the past decade, advances in high-throughput proteomics and interactomics technologies resulted in an accumulation of numerous predicted plant PPIs [25], [26], [27].

Despite the importance of resurrection plants as potential model organisms in studies that focus on the improvement of the desiccation stress tolerance of crops [28] and, despite major advances in the interactome field [29], [30], [31], [32], there is still a dearth of literature exploiting PPIs in these plants. In this study, we created interaction networks of differentially expressed *X*. *schlechteri* (formerly called *X. viscosa*) [33] nuclear proteins as well as observing additional related proteins predicted by GeneMANIA. PPIs analysis is important because it leads to the identification of hubs. Many plant proteins identified as hubs play a role in the plant stress response [24]. Specifically, in plant stress response-related networks, hubs are assumed to play an essential role in the signal transduction cascade following stress detection [34].

The hubs participate in many PPIs and have significant central roles in signaling pathways (reviewed in [24]. Plant hubs can be classified into the functional categories of kinases and phosphatases, transcription factors or components of ubiquitin ligase complexes [24]. Other hub proteins include heat shock proteins (HSPs), ribosomal proteins, proteins involved in redox pathways, proteins involved in DNA repair mechanisms and cytoskeleton-interacting proteins that play a role in protecting cells and cellular components, thus making them essential and often conserved [24], [34], [35], [36], [37]. In addition to these hubs, there are other highly interacting hubs that are still uncharacterized or whose functions are not completely understand [24], [38].

## Methods

**Figure 1.**
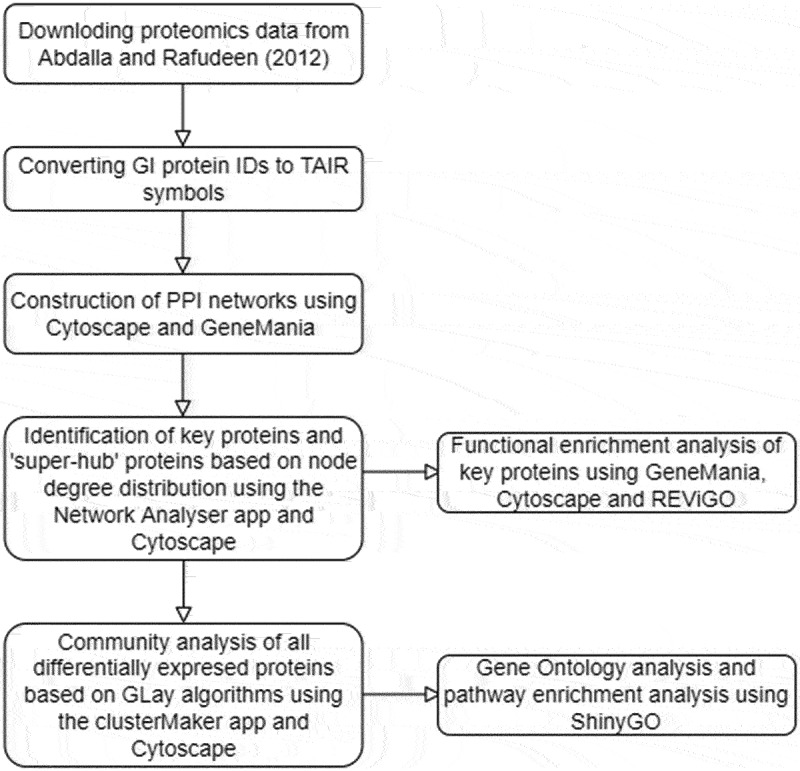
An overview of methodology employed in this study.

A summary of the methodology used in this study is presented in Figure 1.

### Protein data pre-processing

The initial protein list used in PPI analyses was downloaded from the iTRAQ proteomics data from Abdalla and Rafudeen [1]. As far as we are aware, the publication is currently the most comprehensive study on resurrection plant nuclear proteome. The nuclear proteins have a variety of biological functions and are arranged into intricate regulatory networks [39]. Therefore, characterizing the nuclear proteome is crucial for understanding gene regulation in response to desiccation in resurrection plants. The iTRAQ experiment identified 122 proteins with 28 up-regulated, 13 down-regulated and 81 unchanged, in response to desiccation. The GenInfo Identifiers (GI numbers) used for protein IDs in the primary paper were replaced with Arabidopsis Information Resource (TAIR) gene identifiers using the UniProt ID mapping tool (www.uniprot.org) and gProfiler [40]. The conversion to TAIR gene identifiers was necessary to ensure compatibility with the bioinformatics software used in our study. The resulting gene/protein lists are presented in the Appendix (Table S1).

### Construction of PPI networks of DEPs

In order to predict the PPIs between the DEPs, Cytoscape v3.9.0 [41] coupled with the GeneMania v3.5.2 [42] application were used, with *Arabidopsis thaliana* as the source organism. Two interaction networks were constructed using i) up-regulated proteins and ii) down-regulated proteins. The complexity of the networks was reduced by removing duplicate edges and self-loops. The use of GeneMania allows the addition of new genes to the query list that are predicted to be associated with DEPs. Given a query list, GeneMANIA extends the list by adding functionally similar genes that it identifies using available proteomics and genomics data [42], [43] GeneMania also allows the evaluation of relationships of proteins in the respective networks in terms of co-expression, physical and genetic interactions, pathways, colocalization, protein domain similarity, and predicted interactions.

### Identification of Hub proteins

The hub proteins were identified by calculating the node degree distribution by using the Network Analyzer application of Cytoscape [44]. The degree of a node represents the number of edges linked to nodes, and the node with the highest degree represents significant biological functions [20], [45]. A protein with the highest degree distribution was considered as a hub in the respective network.

### Construction of a sub-network consisting of key proteins

The nodes with high degree distribution are viewed as being key to the PPI network [46] and can be considered the most important proteins in the DT mechanism. In this study, for each PPI network, the top 10% of the nodes based on degree distribution were considered as being the key proteins in the response to DT. These key proteins were constructed into a PPI network from which functional enrichment analysis was carried out.

### Functional enrichment analysis of key proteins

In order to obtain deeper insights into the biological reality modeled by the key proteins in each constructed PPI network, we carried out functional enrichment analysis using GeneMania with default settings. Functional enrichment analysis of these key proteins enables a better understanding of the biological relevance of the proteins in response to desiccation stress. GeneMania allowed for the analysis of the statistically overrepresented GO BP (Gene Ontology Biological Process) terms [47]. To reduce the GO term redundancy, REViGO [48] was used. Gene ontology provides a common framework for functionally annotating protein/gene sets [47], [49], [50]. REViGO takes long lists of GO terms and summarizes them by removing the redundant terms. REViGO was implemented with default parameters.

### Community structure analysis to identify functional modules

PPI networks are usually comprised of several sub-networks or functional modules that contribute to various diverse biological processes and pathways. On its own, a node may have negligible influence on the global network or global properties, yet, in a sub-network, it may have a significant influence with specific functionality [51]. Therefore, in a study like ours, it is essential to construct a network consisting of all the DEPs and carry out cluster analysis to identify densely connected regions [51]. Various clustering approaches have been suggested in the literature [52], [53], [54], [55], [56]. In this study, we used community clustering (GLay) [57] to find functional modules (communities) among the DEPs. This was achieved by using Cytoscape coupled with clusterMaker2 v1.3.1 [58]. For community analysis, we constructed a network that was a combination of the up- and down-regulated proteins. To identify the statistically overrepresented GO biological processes within each cluster (communities), the detected clusters were subjected to functional enrichment analyses using the ShinyGO v0.74 tool [59]. The ShinyGO parameters were P-value cutoff (FDR) − 0.05, species - *A. thaliana*, background – all the proteins detected by the proteomics experiment + their predicted associates. For functional enrichment analysis, only communities with at least 10 nodes were analyzed.

## Results and discussion

The role of proteins in different plant biotic and abiotic stress responses is crucial because proteins are the key executors of cellular mechanisms involved in maintaining cellular homeostasis [4]. Also, proteins participate in forming new plant phenotypes to adapt to environmental changes [4]. However, the actions of individual proteins usually do not account for the complex signaling networks and the dynamic regulation of cellular processes involved in the plant response to different stresses [47]. Thus, results from a proteomics experiment may need to be investigated further by such bioinformatics approaches such as PPI studies to obtain a more holistic picture of plant stress response mechanisms. With network biology, it is possible to study the stress response mechanisms in cells by studying the interaction dynamics of the proteins. The interactome may be graphically represented and interpreted by constructing a network in which a protein is represented by a node, while edges represent interactions between the nodes [20]. Generally, biological networks exhibit scale-free properties, where only a few nodes have many connections that represent hubs in the network [24]. In the PPIs network, the nodes with high degrees are defined as the hub proteins [60]. It is widely considered that proteins with high connectivity (hub proteins) play more important biological roles than non-hub proteins, i.e., proteins with low connectivity [45].

### PPI networks of DEPs

**Figure 2.**
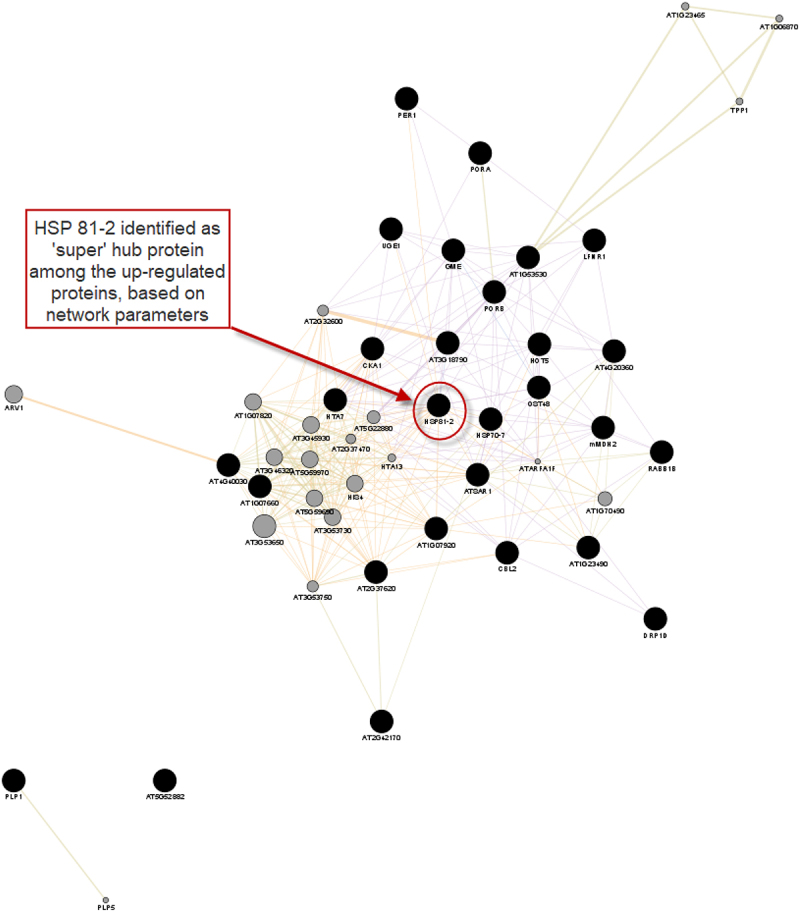
The interaction networks as predicted by GeneMania and visualized in Cytoscape for up-regulated proteins. Black nodes represent query genes while gray nodes represent genes added by GeneMania. The network includes 315 edges between 48 nodes based on co-expression (84.08%), predicted (9.50%), shared protein domains (5.86%) and colocalization (0.55%). Average number of neighbors is 13.956.

**Figure 3.**
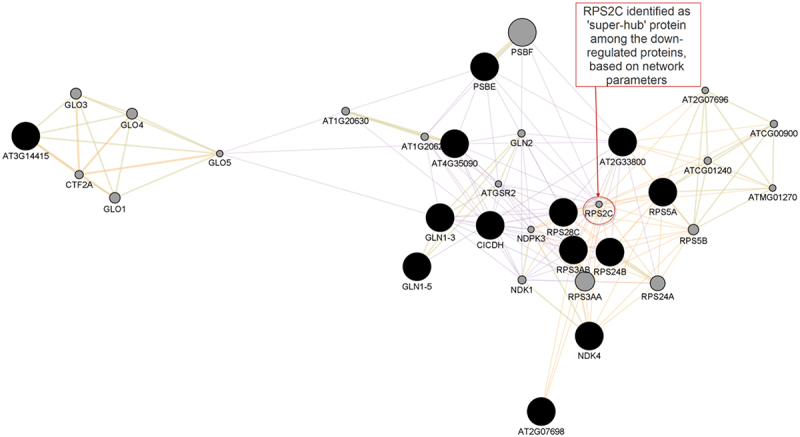
The interaction networks as predicted by GeneMania and visualized in Cytoscape for down-regulated proteins. Black nodes represent query genes while gray nodes represent genes added by GeneMania. The network includes 315 edges between 48 nodes based on co-expression (90.53%), predicted (6.96%), shared protein domains (2.41%) and colocalization (0.10%). Average number of neighbors is 9.879.

An initial set of 28 up-regulated and 13 down-regulated proteins were analyzed in this study. GeneMANIA was used to predict interactions between the DEPs and additional related proteins in the networks, using *A. thaliana* as the source organism. From the up-regulated and down-regulated protein lists, GeneMANIA could recognize additional 20 proteins for both the up-regulated and down-regulated initial query lists. The PPI networks for the DEPs are shown in Figures 2 and 3. It is important to note that the proteins added by GeneMANIA are not necessarily differentially expressed in response to desiccation. Rather, these are proteins predicted to be functionally linked to the DEPs.

In this work, we considered the nodes with the highest degrees distribution as the hub proteins in the PPIs. Using this criterion, the ‘super-hub’ protein for the network constructed from up-regulated proteins, and their predicted associates, was HSP81–2, with a degree distribution of 29. This protein was found in the proteomics experiment among the DEPs. The ‘super-hub’ protein for the down-regulated protein set, and their predicted functionally linked proteins, is RPS2C (40S ribosomal protein S2–3) with a degree distribution of 23. Interestingly, this protein was not in the initial query list but was predicted by GeneMania to be functionally related with the down-regulated proteins.

### Network analysis and functional enrichment analysis of key proteins

In this study, the nodes with a large degree were regarded as ‘key’, and we considered the sub-network of these proteins for further investigation of the key biological processes associated with them. The key proteins (Tables 1 and 2) were subjected to GeneMania to construct sub-networks for both the up-regulated proteins (and their predicted associates) and down-regulated proteins (and their predicted associates). In order to understand the biological processes associated with the key proteins, these proteins were then subjected to network-based functional enrichment analysis in GeneMania.

**Table 1.** List of key proteins among the up-regulated proteins as determined by their node degree distribution.

Gene ID	description	Degree
HSP81–2	Heat shock protein 81–2 [Source:UniProtKB/TrEMBL;Acc:F4K6B6]	29
AT5G22880	Histone H2B [Source:UniProtKB/TrEMBL;Acc:Q1H5F7]	26
HTA13	Probable histone H2A.2 [Source:UniProtKB/Swiss-Prot;Acc:Q9LHQ5]	24
HTA7	Histone H2A [Source:UniProtKB/TrEMBL;Acc:A0A178UFF2]	23
AT2G37470	Histone H2B.4 [Source:UniProtKB/Swiss-Prot;Acc:Q9ZUS0]	21

**Table 2.** Significantly enriched GO biological process terms from the key proteins in the up-regulated proteins and their predicted associates.

GO id	Description	q-value
GO:0045814	negative regulation of gene expression, epigenetic	1.34E–12
GO:0006325	chromatin organization	6.93E–12
GO:0040029	regulation of gene expression, epigenetic	6.93E–12
GO:0034401	chromatin organization involved in regulation of transcription	1.46E–11
GO:0097549	chromatin organization involved in negative regulation of transcription	1.46E–11
GO:0045892	negative regulation of transcription, DNA-templated	5.09E–10
GO:0000785	Chromatin	3.46E–09
GO:0016458	gene silencing	5.00E–09
GO:1903507	negative regulation of nucleic acid-templated transcription	1.48E–07
GO:1902679	negative regulation of RNA biosynthetic process	1.48E–07
GO:0051253	negative regulation of RNA metabolic process	1.80E–07
GO:0045934	negative regulation of nucleobase-containing compound metabolic process	2.25E–07
GO:2000113	negative regulation of cellular macromolecule biosynthetic process	3.66E–07
GO:0010558	negative regulation of macromolecule biosynthetic process	3.66E–07
GO:0000228	nuclear chromosome	5.00E–07
GO:0031647	regulation of protein stability	0.001504
GO:0034728	nucleosome organization	0.009543
GO:0031497	chromatin assembly	0.026893
GO:0065004	protein-DNA complex assembly	0.028496
GO:0006333	chromatin assembly or disassembly	0.028585
GO:0006323	DNA packaging	0.040706
GO:0071824	protein-DNA complex subunit organization	0.070306

#### PPI network of key proteins for the up-regulated proteins and predicted associated proteins

**Figure 4.**
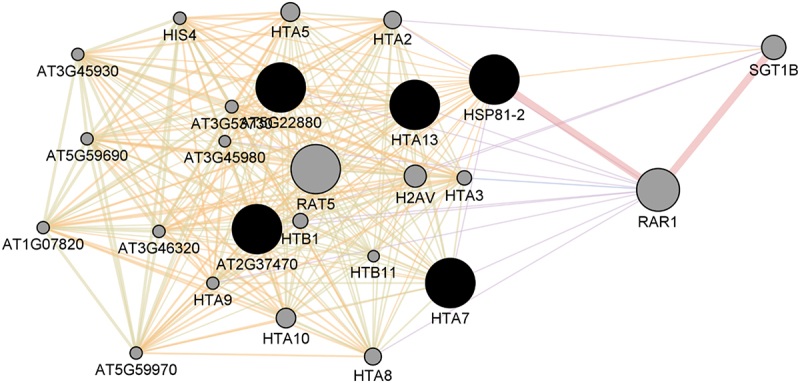
The PPI network of key proteins for up-regulated proteins and the GeneMANIA predicted related proteins. The network includes 203 edges (interactions) between 23 nodes (proteins) based on physical interaction (39.03%), co-expression (38.94%), predicted (16.20%), shared protein domains (3.67%), genetic interaction (1.83) and colocalization (0.33%). Average number of neighbors is 17.652.

HSP81–2, which is the hub protein in the network derived from the up-regulated proteins is also the hub in the corresponding sub-network, with a degree distribution of 29 (Table 1 and Figure 4). Proteins in the HSP family are often identified as hubs in plant network [24]. HSPs or chaperone proteins, are essential for cellular homeostasis and are known to be responsible for proper protein folding localization [61]. After exposure to stressful conditions, such as high temperature and drought, HSPs also direct the abnormal/damaged protein toward the protein degradation machinery for degradation [61], [62], [63], [64], [65], [66]. During drying, cellular contents become concentrated, thereby increasing the likelihood of inappropriate molecular interactions and membrane appression [67]. Ultimately, the lack of sufficient water to surround macromolecules can cause their denaturation and loss of membrane integrity [50], [68], [69]. This loss of macromolecular structural integrity must be mitigated for plant survival during stressful conditions. Desiccation-induced proteins, such as HSPs, are believed to act directly as protective molecules during desiccation and rehydration in resurrection plants [70], [71] Given the critical role of chaperones, it is thus not surprising that HSP81–2, which has chaperonin activity, is a hub in the PPI network of the up-regulated proteins.

The other key proteins in this network are proteins in the histone family. Generally, histones have not been regarded as hubs in PPI networks. However, the fact that histones have been highly interacting in the present work make them interesting targets for future research, especially concerning desiccation stress response. Generally, histones are structural proteins that function to organize eukaryotic DNA [72]. There are four core histones (H2A, H2B, H3, and H4) which are involved in the packaging and maintenance of chromosomes [73], [74] Both histones H2A and H2B play a role in the regulation of transcription and gene expression [72], [75] The fact that we identified these two among the key proteins in our bioinformatics study, suggest that they are important in the DT mechanism in *X*. *schlechteri*. Histone H2B also plays an essential role in DNA replication and repair [72]. Abiotic stresses are known to cause DNA damage in plant cells [76], [77] a situation that compromises cellular integrity and function if the damage is not repaired. Our results, therefore, suggest an important role of H2B in DNA repair in response to desiccation stress in *X*. *schlechteri*.

**Figure 5.**
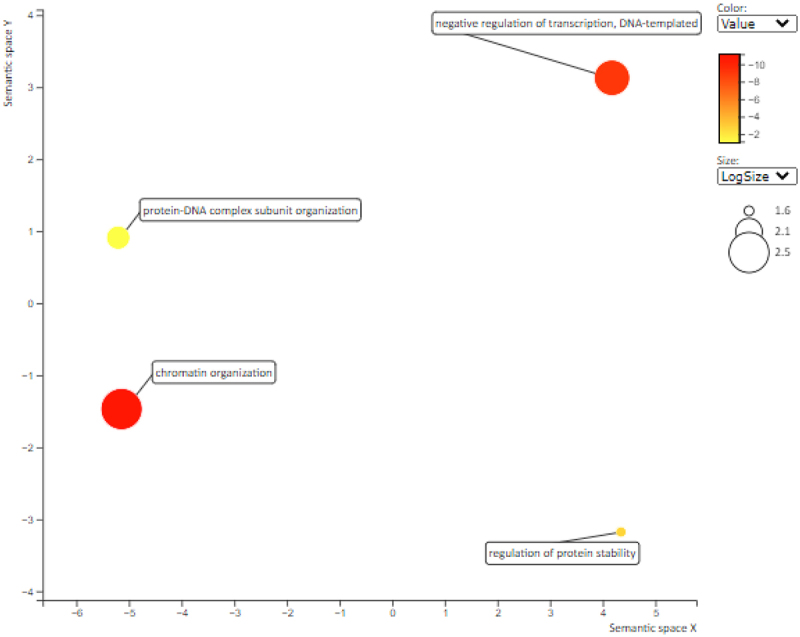
Summary of GO enrichment analysis for the up-regulated key proteins and their predicted related associates.

The overrepresented GO biological process terms include ‘regulation of protein stability’, ‘chromatin organization’ and ‘negative regulation of transcription, DNA templeted’ (see Table 2 and Figure 5). The over-representation of these GO biological process terms shows their importance in stressed cells to ensure the maintenance of protein stability during desiccation and the need to ensure the minimization of synthesis of proteins that are not essential for survival. The minimization of transcription in non-essential processes such as growth and reproduction means that resources are directed toward synthesis of protectant proteins such as antioxidants and chaperonins [6], [21].

Generally, plant stress response involves chromatin re-organization (reviewed in [78]). Such chromatin re-organization includes changes in the nuclear structure [78]. It has been previously suggested that chromatin modifications may be partly responsible for gene regulatory changes necessary for plant DT [79], [80], [81]. Thus, it is not surprising that the GO term ‘chromatin organization’ is overrepresented in proteins from up-regulated proteins network, as this points to the need to ensure the maintenance of chromatin integrity during desiccation stress.

#### PPI network of key proteins for the down-regulated proteins and predicted associated proteins

**Figure 6.**
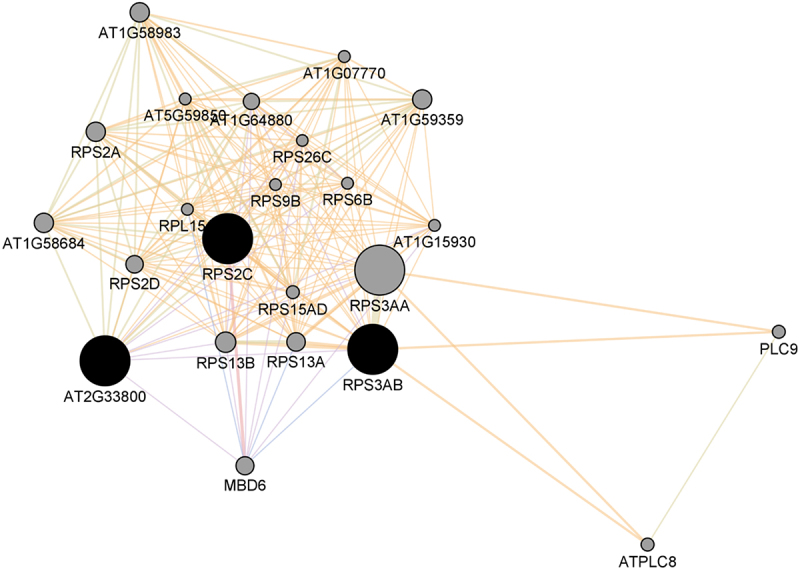
The PPI network of key proteins for down-regulated proteins and the GeneMANIA predicted related proteins. The network includes 269 edges (interactions) between 25 nodes (proteins) based on physical interaction (39.03%), co-expression (38.94%), predicted (16.20%), shared protein domains (3.67%), genetic interaction (1.83) and colocalization (0.33%). Average number of neighbors is 21.520.

**Figure 7.**
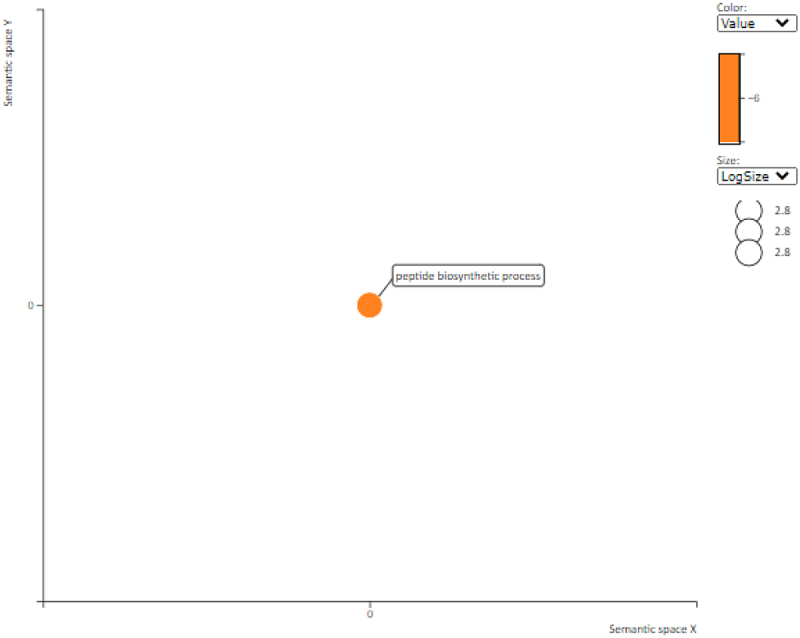
Summary of GO enrichment analysis for the down-regulated key proteins and their predicted related associates.

The key proteins in this PPI network are 30S ribosomal protein S5, chloroplastic and 40S ribosomal protein S3a (Figure 6 and Table 3). Generally, ribosomal proteins have been recognized as hubs in plant PPI networks [24]. Functional enrichment analysis shows that the enriched GO biological process terms included ‘peptide biosynthetic processes’ (Table 4 and Figure 7). These data suggest a general shutting down of translation and protein synthesis during desiccation stress. Water deficit stress has been observed to result in a very significant decrease in global translation rates [82], [83] The apparent down-regulation of processes that are associated with protein synthesis is in agreement with the suggestion that plant development processes often need to be down-regulated to give room to processes involved in protection mechanisms, such as antioxidants and chaperones. In the resurrection plants *Craterostigma plantagineum* and *Haberlea rhodopensis*, water deprivation leads to down-regulation of photosynthesis-related transcripts, but up-regulation of transcripts encoding protective molecules such as late embryogenesis abundant proteins, sucrose, and lipocalins, among others (reviewed in [84]).

**Table 3.** List of key proteins among the down-regulated proteins as determined by their node degree distribution.

name	description	Degree
RPS2C	40S ribosomal protein S2–3 [Source:UniProtKB/Swiss-Prot;Acc:P49688]	23
RPS5	30S ribosomal protein S5, chloroplastic [Source:UniProtKB/Swiss-Prot;Acc:P93014]	17
RPS3AB	40S ribosomal protein S3a [Source:UniProtKB/TrEMBL;Acc:A0A178UZT1]	16

**Table 4.** Significantly enriched GO biological process terms from the key proteins in the down-regulated proteins and their predicted associates.

GO id	Description	q-value
GO:0015935	small ribosomal subunit	1.56E–26
GO:0044391	ribosomal subunit	4.58E–23
GO:0022626	cytosolic ribosome	2.39E–22
GO:0005829	cytosol	2.29E–20
GO:0005198	structural molecule activity	5.03E–11
GO:0043043	peptide biosynthetic process	5.63E–07

## Community structure analysis

**Figure 8.**
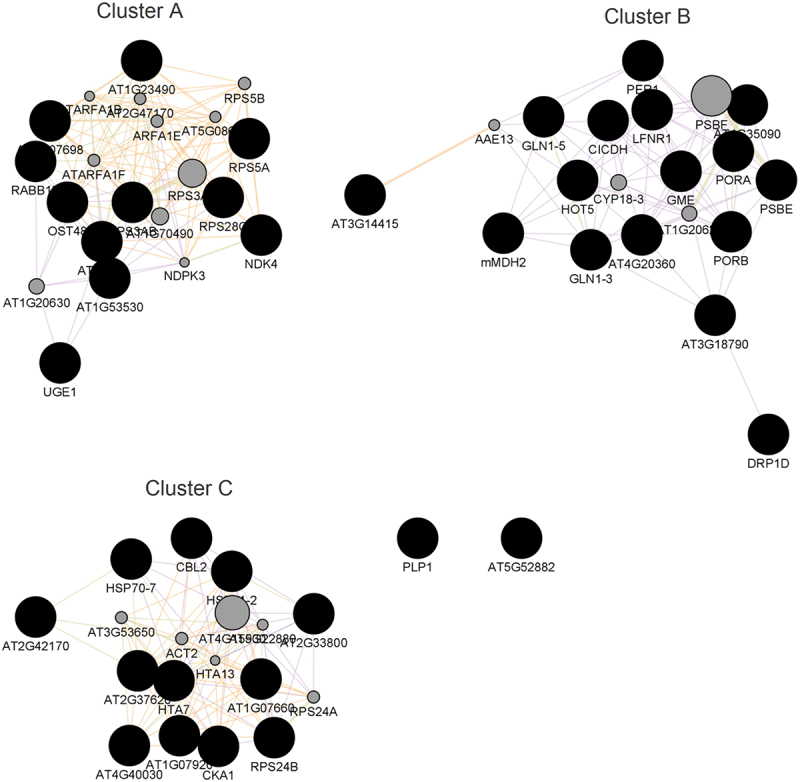
Communities generated by fast greedy (GLay) clustering algorithm are shown.

**Figure 9.**
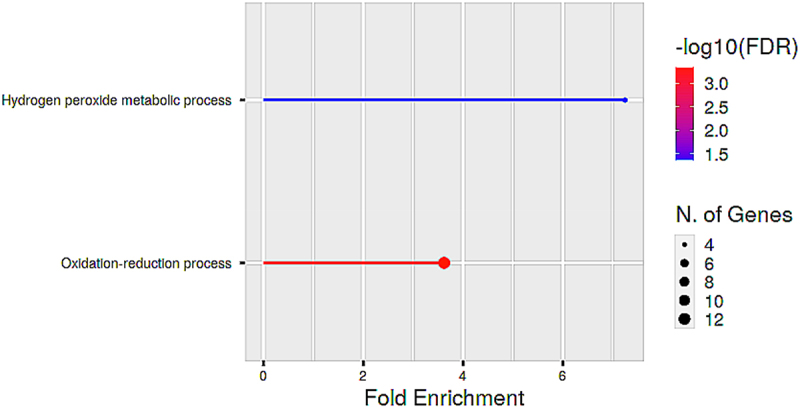
Functional enrichment analysis of proteins in cluster A. The biological processes are ranked by fold enrichment values. The most significant biological processes are shown in red while the less significant processes are highlighted in blue. The larger the dots in the graph, the greater the number of genes involved.

**Figure 10.**
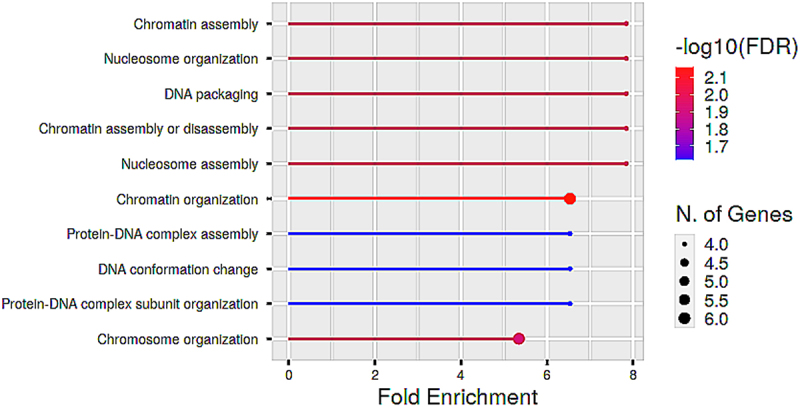
Functional enrichment analysis of proteins in cluster B. The biological processes are ranked by fold enrichment values. The most significant biological processes are shown in red while the less significant processes are highlighted in blue. The larger the dots in the graph, the greater the number of genes involved.

**Figure 11.**
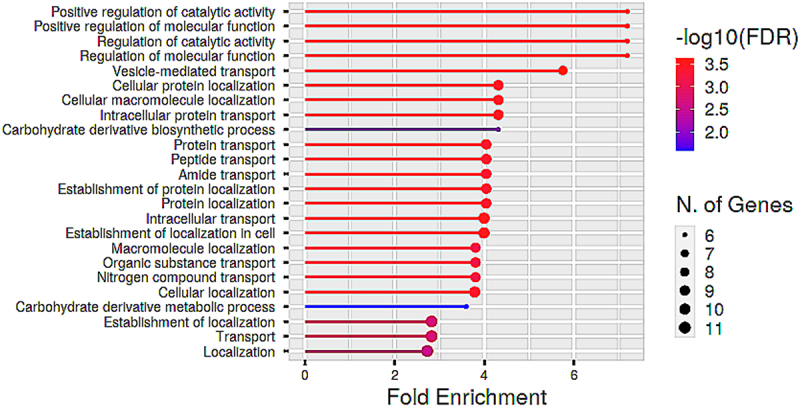
Functional enrichment analysis of proteins in cluster C. The biological processes are ranked by fold enrichment values. The most significant biological processes are shown in red while the less significant processes are highlighted in blue. The larger the dots in the graph, the greater the number of genes involved.

The purpose of community detection for PPI networks is to divide proteins into groups so that the proteins within each group are more similar to one another than those in the other groups [57], [85], [86]. Finding communities is very important, because the communities can reflect functional modules i.e. functional units within a system [86]. Modules are generally regarded as self-contained components of a system with well-defined interfaces with the other components [87]. Topological studies of PPIs can, therefore, help detect and define these modules [87]. In this work, community clustering studies on all DEPs resulted in three protein clusters that had more than 10 members each (Figure 8). We then subjected each cluster to a functional enrichment analysis with the results shown in Figures 9–11.

Cluster A constituted of 21 proteins. Of these, 11 came from the down-regulated proteins, while 8 came from the up-regulated proteins and the remainder were those predicted by GeneMana. The fact that proteins from the up-regulated set and those from the down-regulated set act together as a functional unit suggest a high level of crosstalk within the cell in response to desiccation. The overrepresented GO biological processes in this cluster are those associated with redox processes (Figure 9). The process of desiccation results in the accumulation of reactive oxygen species (ROS) in cells [88]. The accumulation of ROS causes damage to proteins, nucleic acids, lipids, membranes and organelles [22], [89]. Thus, the involvement of proteins associated with anti-oxidant activities is crucial to the survival of the plants. Studies in *X. schlechteri* leaves have shown that peroxidases are up-regulated during the dehydration process and down-regulated upon rehydration [6].

Cluster B consisted of 21 proteins (8 from up-regulated proteins and 11 from down-regulated proteins). This cluster is involved in chromatin organization. The importance of maintaining chromatin integrity has been discussed in the section on ‘PPI network of key proteins for the up-regulated proteins and predicted associated proteins’.

Cluster C consisted of 17 proteins, of which 12 are from the up-regulated proteins. This cluster contains proteins with diverse metabolic processes that can be broadly classified into i) proteins involved in transport and localization, ii) regulation of metabolic processes and iii) proteins involved in carbohydrate metabolism. Generally, this cluster reflects the biological processes involved in the mobilization of resources that is required to get a co-ordinated response against desiccation stress.

## Conclusion

Currently, proteomics studies to understand the mechanisms of DT in resurrection plants have generated a considerable amount of data on the DEPs. However, only a few studies have carried out a thorough investigation using bioinformatics approaches. Mining these DEPs and constructing PPI networks could be regarded as an effective way to explore the biological significance of the DEPs. Firstly, building networks can result in the identification of associated proteins that are otherwise not detected in a conventional proteomics investigation. This is especially important in plant proteomics involving ‘orphan species’ where lists of identified proteins are usually small owing to the lack of specific databases for protein identification. The networks we constructed were all characterized by a small number of highly connected proteins (nodes), while the majority of nodes have only a few connections, which is the classical characteristic of PPI networks [90].

We have used bioinformatics analysis to get more insights from the nuclear proteomics data presented by Abdalla and Rafudeen [1]. The new data produced by our study include the identification of hub proteins in the up-regulated and down-regulated protein sets. Specifically, we showed that the hub proteins for the up-regulated genes were HSP81–2 while 40S ribosomal protein S2–3 was the hub for the down-regulated proteins. Second, we identified functional modules for the DEPs. These functional modules can be grouped into ‘redox proteins’, ‘chromatin organization’ and ‘general metabolism’. Overall, our work suggests that in order to ensure the survival of the plant during desiccation, *X. schlechteri* increases the synthesis of protectant proteins while minimizing the synthesis of non-essential proteins. Our work represents the first effort to use networks biology to understand the mechanisms of DT in resurrection plants.

Understanding the cellular mechanisms of DT in resurrection plants may enable future molecular improvement of drought tolerance in crop plants. Thus, resurrection plants are potential sources for gene discovery. The identification of hub proteins/genes, especially from the up-regulated protein list, can be a potential source for gene discovery. We, however, note that, network biology is meant to be a hypothesis generation process [91], [92], [93]. Thus, the results presented in this study need to be validated experimentally for the predicted interaction networks and the resulting bioinformatics inference to be considered as factual. For example, the predicted PPIs in the results are not from organ (leaf) specific databases and cannot be expected to be 100% accurate. Also, PPI analysis in non-model organisms involves inferences based on organisms such as *A. thaliana*, which are often evolutionarily distantly related. This is an inherent problem associated with bioinformatics of orphan species, such as resurrection plants, whose genomes are either not fully sequenced or are not fully annotated. The primary proteomics experiment identified only a few proteins (122), whose PPIs were analyzed in our work. Potentially, if thousands of proteins could be identified in a proteomics study, additional or new hub proteins and pathways can be identified. Given these limitations, our work can only be considered as a first pilot study for future analysis.

## Data Availability

.The authors confirm that the data supporting the findings of this study are available within the article and the Appendix.

## Appendix

**Table S1.** List of the 122 proteins identified in the iTRAQ experiment by Abdulla and Rafudeen^1^. The GI identifiers from the primary paper were converted to TAIR identifiers to facilitate bioinformatics analysis.

GI identifier	TAIR IDs	description
gi|968977	AT4G27440	Protochlorophyllide reductase B, chloroplastic [Source:UniProtKB/Swiss-Prot;Acc:P21218]
gi|7270538	AT4G35860	Ras-related protein RABB 1b [Source:UniProtKB/Swiss-Prot;Acc:Q38922]
gi|68566307	AT2G44590	At2g44590 [Source:UniProtKB/TrEMBL;Acc:B5×4Z5]
gi|79332762	AT5G67380	Casein kinase II subunit alpha-1 [Source:UniProtKB/Swiss-Prot;Acc:Q08467]
gi|8698725	AT1G12780	Bifunctional UDP-glucose 4-epimerase and UDP-xylose 4-epimerase 1 [Source:UniProtKB/Swiss-Prot;Acc:Q42605]
gi|968975	AT5G54190	NADPH-protochlorophyllide oxidoreductase [Source:UniProtKB/TrEMBL;Acc:A0A178UR02]
gi|21553942	AT5G66680	Dolichyl-diphosphooligosaccharide – protein glycosyltransferase 48 kDa subunit [Source:UniProtKB/Swiss-Prot;Acc:Q944K2]
gi|8778528	AT1G48130	1-Cys peroxiredoxin PER1 [Source:UniProtKB/Swiss-Prot;Acc:O04005]
gi|9758619	AT5G55990	Calcineurin B-like protein 2 [Source:UniProtKB/Swiss-Prot;Acc:Q8LAS7]
gi|145334803	AT5G52882	P-loop containing nucleoside triphosphate hydrolases superfamily protein [Source:UniProtKB/TrEMBL;Acc:F4KHN5]
gi|8671873	AT1G53530	At1g53530 [Source:UniProtKB/TrEMBL;Acc:Q6NLT8]
gi|20465661	AT5G66190	Ferredoxin – NADP reductase, leaf isozyme 1, chloroplastic [Source:UniProtKB/Swiss-Prot;Acc:Q9FKW6]
gi|83754659	AT5G28840	GDP-mannose 3,5-epimerase [Source:UniProtKB/Swiss-Prot;Acc:Q93VR3]
gi|3249104	AT1G09180	Putative GTP-binding protein, SAR1B [Source:UniProtKB/TrEMBL;Acc:O80489]
gi|9758623	AT5G56030	Heat shock protein 81–2 [Source:UniProtKB/TrEMBL;Acc:F4K6B6]
gi|6746592	AT5G49910	Heat shock 70 kDa protein 7, chloroplastic [Source:UniProtKB/Swiss-Prot;Acc:Q9LTX9]
gi|7268831	AT4G20360	Elongation factor Tu, chloroplastic [Source:UniProtKB/Swiss-Prot;Acc:P17745]
gi|145332399	AT3G15020	Malate dehydrogenase 2, mitochondrial [Source:UniProtKB/Swiss-Prot;Acc:Q9LKA3]
gi|15081765	AT1G07920	Elongation factor 1-alpha 4 [Source:UniProtKB/Swiss-Prot;Acc:Q8GTY0]
gi|9758553	AT5G43940	S-(hydroxymethyl)glutathione dehydrogenase [Source:UniProtKB/TrEMBL;Acc:F4K7D6]
gi|30688915	AT2G42170	Actin family protein [Source:UniProtKB/TrEMBL;Acc:Q8GWA5]
gi|9758835	AT1G07660	Histone H4 [Source:UniProtKB/Swiss-Prot;Acc:P59259]
gi|79324605	AT2G37620	Actin-3 [Source:UniProtKB/Swiss-Prot;Acc:P0CJ47]
gi|145334271	AT4G40030	Histone superfamily protein [Source:UniProtKB/TrEMBL;Acc:A8MRL0]
gi|75306451	AT5G27670	Histone H2A [Source:UniProtKB/TrEMBL;Acc:A0A178UFF2]
gi|9293904	AT3G18790	AT3G18790 protein [Source:UniProtKB/TrEMBL;Acc:Q9LS97]
gi|8778579	AT1G23490	ADP-ribosylation factor 2-B [Source:UniProtKB/Swiss-Prot;Acc:P0DH91]
gi|7270656	AT4G37070	Patatin [Source:UniProtKB/TrEMBL;Acc:A0A178UVZ8]
Unchanged		
gi|7019676	AT3G59820	LETM1-like protein [Source:UniProtKB/TrEMBL;Acc:F4J9G6]
gi|8777330	AT5G58290	RPT3 [Source:UniProtKB/TrEMBL;Acc:A0A178UAH2]
gi|15241777	AT5G39500	ARF guanine-nucleotide exchange factor GNL1 [Source:UniProtKB/Swiss-Prot;Acc:Q9FLY5]
gi|601843	AT2G45820	Remorin [Source:UniProtKB/Swiss-Prot;Acc:O80837]
gi|6721173	AT3G04120	Glyceraldehyde-3-phosphate dehydrogenase GAPC1, cytosolic [Source:UniProtKB/Swiss-Prot;Acc:P25858]
gi|9955568	AT5G04130	DNA gyrase subunit B, mitochondrial [Source:UniProtKB/Swiss-Prot;Acc:Q94BZ7]
gi|9758113	AT5G55160	Small ubiquitin-related modifier [Source:UniProtKB/TrEMBL;Acc:F4K3D6]
gi|7270856	AT4G38740	Peptidyl-prolyl cis-trans isomerase [Source:UniProtKB/TrEMBL;Acc:A0A178V2S8]
gi|23198084	AT1G56190	Phosphoglycerate kinase [Source:UniProtKB/TrEMBL;Acc:A0A178W4Q1]
gi|5306242	AT2G15910	CSL zinc finger domain-containing protein [Source:UniProtKB/TrEMBL;Acc:Q9×IM2]
gi|75276926	AT1G08880	Histone H2A [Source:UniProtKB/TrEMBL;Acc:A0A178W8H7]
gi|231683	AT5G61790	Calnexin homolog 1 [Source:UniProtKB/Swiss-Prot;Acc:P29402]
gi|7529717	AT3G52930	Fructose-bisphosphate aldolase [Source:UniProtKB/TrEMBL;Acc:A0A178V8L4]
gi|8778615	AT1G20580	Small nuclear ribonucleoprotein SmD3b [Source:UniProtKB/Swiss-Prot;Acc:Q9LM92]
gi|9758940	AT5G45930	Magnesium-chelatase subunit ChlI-2, chloroplastic [Source:UniProtKB/Swiss-Prot;Acc:Q5×F33]
gi|9279611	AT3G26060	Thioredoxin superfamily protein [Source:UniProtKB/TrEMBL;Acc:F4JBC9]
gi|9755651	AT5G16050	14-3-3-like protein GF14 upsilon [Source:UniProtKB/Swiss-Prot;Acc:P42645]
gi|9759623	AT5G65430	General regulatory factor 8 [Source:UniProtKB/TrEMBL;Acc:F4KHY7]
gi|75313476	AT2G38810	Probable histone H2A variant 2 [Source:UniProtKB/Swiss-Prot;Acc:Q9SII0]
gi|7576225	AT3G48870	Chaperone protein ClpC2, chloroplastic [Source:UniProtKB/Swiss-Prot;Acc:Q9S×J7]
gi|98960893	AT5G59910	HTB4 [Source:UniProtKB/TrEMBL;Acc:A0A384KCB6]
gi|73921087	AT2G34160	Uncharacterized protein At2g34160 [Source:UniProtKB/Swiss-Prot;Acc:O22969]
gi|79314743	AT3G51260	Proteasome subunit alpha type [Source:UniProtKB/TrEMBL;Acc:A0A178VPE4]
gi|7630068	AT3G58730	V-type proton ATPase subunit D [Source:UniProtKB/Swiss-Prot;Acc:Q9×GM1]
gi|6714442	AT3G05680	Protein virilizer homolog [Source:UniProtKB/Swiss-Prot;Acc:F4J8G7]
gi|9757906	AT1G07770	40S ribosomal protein S15a-1 [Source:UniProtKB/Swiss-Prot;Acc:P42798]
gi|6692094	AT1G64200	vacuolar H±ATPase subunit E isoform 3 [Source:TAIR;Acc:AT1G64200]
gi|75335836	AT3G58570	DEAD-box ATP-dependent RNA helicase 52 [Source:UniProtKB/Swiss-Prot;Acc:Q9M2F9]
gi|22022513	AT5G42270	VAR1 [Source:UniProtKB/TrEMBL;Acc:A0A178U8J6]
gi|6899900	AT3G62020	At3g62020 [Source:UniProtKB/TrEMBL;Acc:A0JQ09]
gi|8978263	AT5G49665	E3 ubiquitin-protein ligase WAV3 [Source:UniProtKB/Swiss-Prot;Acc:Q9LTA6]
gi|6721109	AT1G76030	V-type proton ATPase subunit B1 [Source:UniProtKB/Swiss-Prot;Acc:P11574]
gi|79313181	AT3G10920	Superoxide dismutase [Mn] 1, mitochondrial [Source:UniProtKB/Swiss-Prot;Acc:O81235]
gi|26454661	AT3G61860	RSP31 [Source:UniProtKB/TrEMBL;Acc:A0A178VG60]
gi|7576200	AT3G60245	60S ribosomal protein L37a-2 [Source:UniProtKB/Swiss-Prot;Acc:Q8RXU5]
gi|9757970	AT5G37600	Glutamine synthetase cytosolic isozyme 1–1 [Source:UniProtKB/Swiss-Prot;Acc:Q56WN1]
gi|125662843	AT1G08830	Superoxide dismutase [Cu-Zn] 1 [Source:UniProtKB/Swiss-Prot;Acc:P24704]
gi|3776021	AT1G72730	Eukaryotic initiation factor 4A–3 [Source:UniProtKB/Swiss-Prot;Acc:Q9CAI7]
gi|9755742	AT5G14740	Beta carbonic anhydrase 2, chloroplastic [Source:UniProtKB/Swiss-Prot;Acc:P42737]
gi|892133231	AT2G38670	Ethanolamine-phosphate cytidylyltransferase [Source:UniProtKB/Swiss-Prot;Acc:Q9ZVI9]
gi|8698736	AT1G12900	glyceraldehyde 3-phosphate dehydrogenase A subunit 2 [Source:TAIR;Acc:AT1G12900]
gi|3776005	AT5G11170	DEAD-box ATP-dependent RNA helicase 56 [Source:UniProtKB/Swiss-Prot;Acc:Q9LFN6]
gi|28436479	AT5G20620	Polyubiquitin 4 [Source:UniProtKB/Swiss-Prot;Acc:P0CH32]
gi|7671467	AT5G10360	40S ribosomal protein S6 [Source:UniProtKB/TrEMBL;Acc:A0A178USM6]
gi|4204274	AT1G67090	Ribulose bisphosphate carboxylase small chain [Source:UniProtKB/TrEMBL;Acc:A0A178WD57]
gi|30681260	AT3G10350	P-loop containing nucleoside triphosphate hydrolases superfamily protein [Source:UniProtKB/TrEMBL;Acc:F4J3Q8]
gi|6686407	AT1G65710	Uncharacterized protein At1g65710 [Source:UniProtKB/Swiss-Prot;Acc:Q5×VH5]
gi|17939849	AT5G08690	ATP synthase subunit beta-2, mitochondrial [Source:UniProtKB/Swiss-Prot;Acc:P83484]
gi|4586035	AT2G20530	Prohibitin-6, mitochondrial [Source:UniProtKB/Swiss-Prot;Acc:Q9SIL6]
gi|9294382	AT3G14600	60S ribosomal protein L18a-3 [Source:UniProtKB/Swiss-Prot;Acc:Q9LUD4]
gi|9294373	AT3G12580	Probable mediator of RNA polymerase II transcription subunit 37c [Source:UniProtKB/Swiss-Prot;Acc:Q9LHA8]
gi|21537149	AT3G49010	60S ribosomal protein L13–1 [Source:UniProtKB/Swiss-Prot;Acc:P41127]
gi2565436	At3g27925	DegP protease precursor
gi|24030464	AT5G17170	Rubredoxin family protein [Source:UniProtKB/TrEMBL;Acc:Q9FFJ2]
gi|7525046	ATCG00540	Cytochrome f [Source:UniProtKB/Swiss-Prot;Acc:P56771]
gi|9758504	AT5G46680	Pentatricopeptide repeat-containing protein At5g46680 [Source:UniProtKB/Swiss-Prot;Acc:Q56×R6]
gi|2232146	AT1G35160	GF14 protein phi chain [Source:UniProtKB/TrEMBL;Acc:F4HWQ5]
gi|9758239	AT5G50920	Chaperone protein ClpC1, chloroplastic [Source:UniProtKB/Swiss-Prot;Acc:Q9FI56]
gi|806279	AT1G61580	60S ribosomal protein L3–2 [Source:UniProtKB/Swiss-Prot;Acc:P22738]
gi|68566309	AT1G10290	DRP2A [Source:UniProtKB/TrEMBL;Acc:A0A178WBB3]
gi|145339693	AT3G60100	Citrate synthase [Source:UniProtKB/TrEMBL;Acc:A0A1I9LSJ6]
gi|79321468	AT1G78900	VHA-A [Source:UniProtKB/TrEMBL;Acc:A0A384LM33]
gi|7525018	ATCG00120	ATP synthase subunit alpha, chloroplastic [Source:UniProtKB/TrEMBL;Acc:A0A1B1W4S8]
gi|2102657	AT1G72370	40S ribosomal protein SA [Source:UniProtKB/TrEMBL;Acc:A0A178WNA1]
gi|9759565	AT5G07090	40S ribosomal protein S4–2 [Source:UniProtKB/Swiss-Prot;Acc:P49204]
gi|87116612	AT4G09000	General regulatory factor 1 [Source:UniProtKB/TrEMBL;Acc:F4JJ94]
gi|7269769	AT4G02840	[Source:UniProtKB/TrEMBL;Acc: A0A178V1I1]
gi|41019517	ATMG00070	NADH dehydrogenase subunit 9 [Source:TAIR;Acc:ATMG00070]
gi|4406816	AT2G18020	EMB2296 [Source:UniProtKB/TrEMBL;Acc:A0A178VSS0]
gi|21553582	AT5G40950	50S ribosomal protein L27, chloroplastic [Source:UniProtKB/Swiss-Prot;Acc:Q9FLN4]
gi|27808637	AT3G60770	40S ribosomal protein S13–1 [Source:UniProtKB/Swiss-Prot;Acc:P59223]
gi|98960873	AT3G09680	40S ribosomal protein S23–1 [Source:UniProtKB/Swiss-Prot;Acc:Q9SF35]
gi|84028256	AT5G52650	40S ribosomal protein S10–3 [Source:UniProtKB/Swiss-Prot;Acc:Q9LTF2]
gi|4218014	AT2G18510	Emb2444 [Source:UniProtKB/TrEMBL;Acc:A0A178VZK5]
gi|90568002	AT5G39850	40S ribosomal protein S9–2 [Source:UniProtKB/Swiss-Prot;Acc:Q9FLF0]
gi|79322198	AT2G05840	Proteasome subunit alpha type-6-B [Source:UniProtKB/Swiss-Prot;Acc:O81147]
gi|30680605	AT1G08360	60S ribosomal protein L10a-1 [Source:UniProtKB/Swiss-Prot;Acc:Q8VZB9]
gi|71151986	AT5G13850	Nascent polypeptide-associated complex subunit alpha-like protein 3 [Source:UniProtKB/Swiss-Prot;Acc:Q6ICZ8]
gi|9798392	AT3G44890	RPL9 [Source:UniProtKB/TrEMBL;Acc:A0A178VBQ7]
gi|9758455	AT5G04800	40S ribosomal protein S17–4 [Source:UniProtKB/Swiss-Prot;Acc:Q9LZ17]
gi|7525041	ATCG00490	Ribulose bisphosphate carboxylase large chain [Source:UniProtKB/Swiss-Prot;Acc:O03042]
gi|7270417	AT4G34670	40S ribosomal protein S3a [Source:UniProtKB/TrEMBL;Acc:A0A178UZT1]
gi|75101015	AT2G33800	30S ribosomal protein S5, chloroplastic [Source:UniProtKB/Swiss-Prot;Acc:P93014]
gi|79324564	AT2G37270	40S ribosomal protein S5–1 [Source:UniProtKB/Swiss-Prot;Acc:Q9ZUT9]
gi|8778687	AT1G48470	Glutamine synthetase cytosolic isozyme 1–5 [Source:UniProtKB/Swiss-Prot;Acc:Q8G×W5]
gi|28393681	AT3G17820	Glutamine synthetase [Source:UniProtKB/TrEMBL;Acc:A0A178VNQ6]
gi|7270460	AT4G35090	catalase 2 [Source:TAIR;Acc:AT4G35090]
gi|20198280	AT2G07698	ATPase, F1 complex, alpha subunit protein [Source:UniProtKB/TrEMBL;Acc:F4IMB5]
gi|464720	AT5G64140	RPS28 [Source:UniProtKB/TrEMBL;Acc:A0A178UA91]
gi|21555157	AT5G28060	40S ribosomal protein S24–2 [Source:UniProtKB/Swiss-Prot;Acc:Q8LC83]
gi|7269239	AT4G23900	Nucleoside diphosphate kinase IV, chloroplastic/mitochondrial [Source:UniProtKB/Swiss-Prot;Acc:Q8LAH8]
gi|6227018	AT1G65930	Isocitrate dehydrogenase [NADP] [Source:UniProtKB/TrEMBL;Acc:A0A178W7K0]
gi|62320779	AT3G14415	GOX2 [Source:UniProtKB/TrEMBL;Acc:A0A384L6×3]
gi|114152861	ATCG00580	Cytochrome b559 subunit alpha [Source:UniProtKB/TrEMBL;Acc:A0A1B1W4W2]
